# Effect of Age and Biological Subtype on the Risk and Timing of Brain Metastasis in Breast Cancer Patients

**DOI:** 10.1371/journal.pone.0089389

**Published:** 2014-02-24

**Authors:** Man-Hsin Hung, Chun-Yu Liu, Cheng-Ying Shiau, Chin-Yi Hsu, Yi-Fang Tsai, Yu-Ling Wang, Ling-Chen Tai, Kuang-Liang King, Ta-Chung Chao, Jen-Hwey Chiu, Cheng-Hsi Su, Su-Shun Lo, Cheng-Hwai Tzeng, Yi-Ming Shyr, Ling-Ming Tseng

**Affiliations:** 1 Division of Hematology and Oncology, Department of Medicine, Taipei Veterans General Hospital, Taipei, Taiwan; 2 Division of General Surgery, Department of Surgery, Taipei Veterans General Hospital, Taipei, Taiwan; 3 Cancer Center, Taipei Veterans General Hospital, Taipei, Taiwan; 4 School of Medicine, National Yang-Ming University, Taipei, Taiwan; 5 Institute of Biopharmaceutical Sciences, National Yang-Ming University, Taipei, Taiwan; 6 Program in Molecular Medicine, School of Life Sciences, National Yang-Ming University, Taipei, Taiwan; 7 Department of Pathology and Laboratory Medicine, Taipei Veterans General Hospital, Taipei, Taiwan; 8 Institute of Traditional Medicine, School of Medicine, National Yang-Ming University, Taipei, Taiwan; 9 Division of General Surgery, Department of Surgery, Cheng Hsin General Hospital, Taipei, Taiwan; 10 National Yang-Ming University Hospital, I-Lan, Taiwan; Stanford University School of Medicine, United States of America

## Abstract

**Background:**

Brain metastasis is a major complication of breast cancer. This study aimed to analyze the effect of age and biological subtype on the risk and timing of brain metastasis in breast cancer patients.

**Patients and Methods:**

We identified subtypes of invasive ductal carcinoma of the breast by determining estrogen receptor, progesterone receptor and HER2 status. Time to brain metastasis according to age and cancer subtype was analyzed by Cox proportional hazard analysis.

**Results:**

Of the 2248 eligible patients, 164 (7.3%) developed brain metastasis over a median follow-up of 54.2 months. Age 35 or younger, HER2-enriched subtype, and triple-negative breast cancer were significant risk factors of brain metastasis. Among patients aged 35 or younger, the risk of brain metastasis was independent of biological subtype (*P = *0.507). Among patients aged 36–59 or >60 years, those with triple-negative or HER2-enriched subtypes had consistently increased risk of brain metastasis, as compared with those with luminal A tumors. Patients with luminal B tumors had higher risk of brain metastasis than luminal A only in patients >60 years.

**Conclusions:**

Breast cancer subtypes are associated with differing risks of brain metastasis among different age groups. Patients age 35 or younger are particularly at risk of brain metastasis independent of biological subtype.

## Introduction

Brain metastasis is a major complication of breast cancer that significantly impairs patients’ survival and quality of life [1], [2]. It occurs in 3–6% of all breast cancer patients [3], and the risk can increase to 10–43% in patients with metastatic breast cancer [4]–[6]. Despite advances in treatment and supportive care, outcomes in breast cancer patients with brain metastasis remain poor. The median survival after brain metastasis is 3–9 months [4], [6]. Therefore, identification of patients at higher risk for brain metastasis is needed to develop appropriate detection and even prevention strategies for these patients.

Several risk factors for brain metastasis have been reported of which age is the most commonly documented [4], [7], [8]. In a retrospective study of 219 patients with metastatic breast cancer, Evans *et al.* reported that the incidence of brain metastasis was significantly higher in patients younger than 40 years old compared to patients older than 60 (43% *vs.* 8%; *P = *0.0002). [8] Similarly, Tsukada Y *et al.* reported that the median age of patients with central nervous system (CNS) metastasis was approximately 5 years younger than patients without [7].

Expression of particular biomarkers represents another major risk factor for brain metastasis. These biomarkers include the estrogen receptor (ER), progesterone receptor (PR), and human epidermal growth factor receptor 2 (HER2) [4]–[6], [8]–[10]. Patients with negative ER expression or with HER2 overexpression have increased incidence of brain metastasis [4]–[7], [9], [10]. This association reflects the inherent heterogeneity of breast cancer. Four different biological subtypes of breast cancer can be defined by results of immunohistochemical (IHC) staining of ER, PR and HER2 [11], [12]. Despite the fact that both age and certain biomarkers being known risk factor for brain metastasis in breast cancer, prior studies have provided limited information regarding the relationship between age and the development of brain metastasis among the subtypes. Therefore, here we aimed to characterize brain metastasis risk according to both age and breast cancer subtype.

## Patients and Methods

### Study Population

Female patients with stage I-IV invasive ductal carcinoma who were diagnosed and treated in Taipei Veteran’s General Hospital between January 2000 and December 2009 were retrospectively identified from the hospital breast cancer database. Patient’s clinical information, diagnosis date, pathological features including histological grade, IHC staining for ER and PR, IHC staining or fluorescence in situ hybridization (FISH) for HER2, subsequent outcome and last follow-up date were collected from chart review. Patients were excluded if they had cancers other than invasive ductal carcinoma, incomplete pathological results, or incomplete follow-ups. This study was approved by the institutional review board of Taipei Veterans’ General Hospital. All the data in this trial were collected during routine clinical care in the hospital and there was no direct contact with patients for any data collection or analysis, as such written consents from study subjects were waived by the institutional review board.

### Classification of Breast Cancer Subtype

All of the tumor tissue was managed and interpreted by specific breast cancer pathologists following the College of American Pathologists Protocol [13], [29]. Positive ER and PR results were defined as IHC staining in more than 10% of cells according to the applicable protocol during the study period. “HER2 positive” was defined as either an IHC staining score of 3+, or 2+ with gene amplification shown by FISH [13]. Histologic grade was assessed by the Nottingham grading system [14]. Because of growing evidence suggesting that histologic grade or Ki-67 status helps discriminate luminal breast cancer subtypes [12], patients were categorized into the following breast cancer subtypes according to the 2011 St. Gallen International Breast Cancer Expert Panel guidelines: luminal A patients were ER- and/or PR-positive, HER2-negative, and histological grade 1 or 2; luminal B patients were ER- and/or PR-positive and HER2-positive, or were ER- and/or PR-positive and histological grade 3; HER2-positive patients were HER2-positive and negative for both ER and PR; and triple-negative breast cancer (TNBC) patients were negative for ER, PR, and HER2 [12].

#### Patient management and surveillance of brain metastasis

All enrolled patients received staging, treatment and follow-up based on the breast cancer treatment guidelines of our institution which are regularly updated, and in accordance with the latest available recommendations of the National Comprehensive Cancer Network [13] and the St. Gallen consensus [12]. Treatment protocols were established on an individual patient basis that considered general performance status, biological subtype, disease status, and other risk factors. Treatment protocols were regularly reviewed by a multidisciplinary team. Hormone therapy was given to patients with hormone-positive disease unless otherwise contraindicated or if there was intolerance. Chemotherapy and radiotherapy were administrated to patients with appropriate indications. For patients with tumors with HER2-overexpression, anti-HER2 treatments such as trastuzumab or lapatinib were used wherever available. Follow-up visits at regular intervals, every month in the first three years and every three months subsequently were arranged for every patient. Imaging such as computed tomography (CT) of chest, bone scan, and others were performed every three months or longer as clinically needed. Brain metastasis was confirmed by either CT scan or magnetic resonance imaging (MRI) that was performed whenever CNS metastasis was suspected clinically. Brain metastases were treated both locally (e.g., by radiotherapy and surgery) and systemically (e.g., by chemotherapy), according to the overall clinical condition and tolerance of each patient.

### Statistical Analysis

To determine the impact of age, we divided patient’s age at diagnosis into three groups according to a previous study: ≤35 years, 36–59 years, and ≥60 years [15]. Descriptive statistical analysis was used to compare baseline characteristics among different age groups and breast cancer subtypes. Continuous data were summarized by using medians and interquartile ranges (IQRs). Categorical data were summarized using counts and percentages. Time to brain metastasis was defined as the time between initial breast cancer diagnosis and the date of brain metastasis confirmed by image study. Overall survival (OS) was calculated from the time of initial breast cancer diagnosis to the date of death or last consultation; OS from brain metastasis was also calculated using the date when brain metastasis was detected to the date of death or last consultation. The Kaplan–Meier method was used to estimate the cumulative incidence of brain metastasis and OS; log-rank tests were used for comparisons. A Cox proportional hazards model was used to analyze the contribution of each possible factor on the time to brain metastasis. Variables with P values of less than 0.10 in univariate analyses were entered into multivariate analyses. A two-sided P value of less than 0.05 was considered statistically significant. All statistical analyses were performed using SPSS software (version 17.0, SPSS, Chicago, IL, USA).

## Results

### General Characteristics of Patients According to Age and Breast Cancer Subtype

There were 2248 patients enrolled in this study. Their general characteristics are summarized in Table 1. Among the subtypes, hormone receptor-positive disease was the most common (69%), followed by TNBC (17.6%) and HER2-enriched disease (13.4%). Most patients were initially treated with surgical resection (97.4%). Chemotherapy was given to 71% (n = 1596) of all patients, while 92.3% (n = 1432) of patients with hormone receptor-positive disease received hormone therapy, and 39.5% (n = 205) of patients with HER2-enriched disease had anti-HER2 treatments. Median follow-up time for all patients was 4.5 years. During the follow-up period, 351 patients (15.6%) had recurrent disease and 243 patients (10.8%) died of breast cancer.

**Table 1:** General characteristics of patient (n = 2248).

Characteristics		Number	%
**Age, year**	≤35	104	4.6
	36–59	1503	66.9
	≥60	641	28.5
**T stage**	T1	905	40.3
	T2	1103	49.1
	T3	129	5.7
	T4	97	4.3
	Unknown	14	0.6
**No. of positive lymph nodes**	0	1264	56.2
	1–3	466	20.8
	4–9	313	13.9
	>9	205	9.1
**Metastatic disease at initial diagnosis**		63	2.8
**Grade**	1	204	9.1
	2	1363	60.6
	3	681	30.3
**ER+**		1476	65.7
**PR+**		1298	57.7
**HER2+** *		517	23.0
**Biological subtypes**	Luminal A	1104	49.1
	Luminal B	448	19.9
	HER2 enriched	301	13.4
	TNBC	395	17.6
**Operation**		2189	97.4
	Breast conserving	1797	
	Mastectomy	392	
**Chemotherapy**		1596	71.0
**Hormone therapy**		1432	63.7
**Radiotherapy**		786	35.0

*HER2+ was defined as either 3+ on IHC staining, or 2+ on IHC staining with gene amplification shown by FISH.

ER: estrogen receptor; PR: progesterone receptor; TNBC: triple-negative breast cancer.

**Figure 1:**
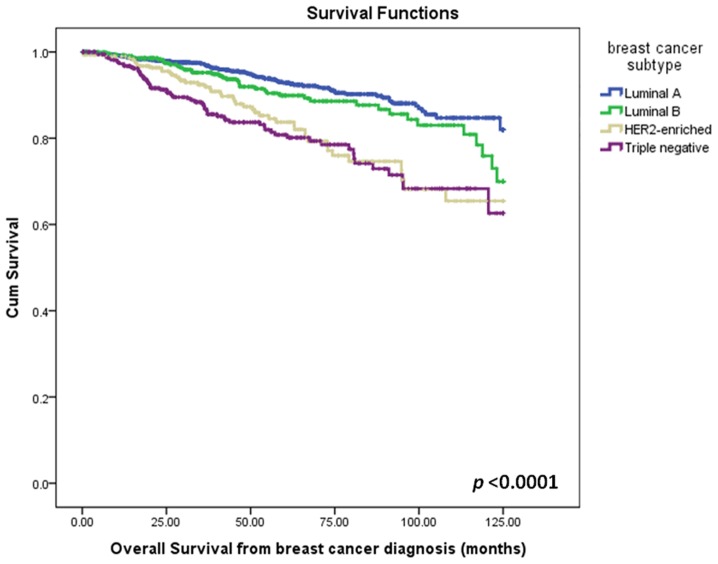
Overall survival from time of diagnosis by breast cancer subtype.

In general, age of diagnosis and surgical resection rate did not differ statistically among the four biological subtypes. However, patients with HER2-enriched disease and TNBC presented with more advanced disease at initial diagnosis (*P*<0.001), had a higher probability of receiving chemotherapy, and had higher mortality rates when compared with patients with luminal A breast cancers (Table 2 and Fig. 1). The estimated 5-year OS was 92.8%, 89.9%, 83.7% and 80.8% in luminal A, luminal B, HER2-enriched and TNBC, respectively.

**Table 2:** General characteristics of patients according to breast cancer subtypes.

Characteristics	Total	Luminal A	Luminal B	HER2–enriched	Triple negative	*P*
	N = 2248	N = 1104	N = 448	N = 301	N = 395	
**Median age (IQR)**	52 (61–45)	51 (61–45)	51 (60–44)	54 (61–47)	53 (61–46)	
**Age group, years (%)**						0.62
≤35	104 (4.6)	47 (4.3)	24 (5.4)	10 (3.3)	23(5.8)	
36–59	1503(66.9)	744 (67.4)	299 (66.7)	202 (67.1)	258 (65.3)	
≥60	641(28.5)	313 (28.4)	125 (27.9)	89 (29.6)	114 (28.9)	
**AJCC stage at initial diagnosis (%)**						<0.001
1	666 (29.6)	411 (37.2)	102 (22.8)	60(19.9)	93 (23.5)	
2	467 (43.0)	447 (40.5)	194 (43.3)	137 (45.5)	190 (48.1)	
3	545 (24.2)	219 (19.8)	141 (31.5)	88 (29.2)	97 (24.6)	
4	63 (2.8)	21 (1.9)	11 (2.5)	15 (5.0)	15 (3.8)	
Unknown	7 (0.3)	6 (0.5)	0	1 (0.3)	0	
**Operation (%)**	2189 (97.4)	1079 (97.7)	443 (98.9)	286 (95.0)	381 (96.5)	0.306
**Chemotherapy (%)**	1596 (71.0)	653 (59.1)	324 (72.3)	265 (88.0)	354 (89.6)	<0.001
**Hormone therapy (%)**	1432 (63.7)	1003 (90.9)	429 (95.8)	0	0	<0.001
**Brain metastasis event (%)**	164 (7.3)	49 (4.4)	33 (7.4)	34 (11.3)	48 (12.2)	<0.001
**Cumulative incidence of brain metastasis,% (95% CI)**						<0.001
1 year	1.2 (1.0–1.4)	0.8 (0.5–1.1)	0.5 (0.2–0.8)	2.5 (1.6–3.4)	1.9 (1.2–2.6)	
3 year	4.9 (4.4–5.4)	2.2 (1.7–2.7)	3.5 (2.5–4.5)	8.3 (6.5–10.1)	11.3 (9.4–13.2)	
5 year	8.2 (7.5–8.9)	3.7 (3.0–4.4)	7.2 (5.6–8.8)	15.7 (12.9–18.5)	15.8 (13.5–18.1)	

AJCC, American Joint Committee on Cancer; CI, confidence interval; IQR, interquartile range; TNBC: triple negative breast cancer.

With respect to age groups, younger patients were more likely to receive chemotherapy (91.3% of patients aged ≤35 years compared with 59.0% of patients aged ≥60 years; Table 3). Estimated 5-year OS was 84.2%, 89.8%, and 86.7% for patients in the age groups ≤35, 36–59, and ≥60, respectively. (*P = *0.056).

**Table 3:** General characteristics of patients with brain metastasis according to age.

Characteristics	Total	Age ≤35	Age 36–59	Age ≥60	*P*
	N = 2248	N = 104	N = 1503	N = 641	
**Breast cancer subtype**					0.767
**Luminal A**	1104 (49.1)	47 (45.2)	744 (49.5)	313 (48.8)	
**Luminal B**	448 (19.9)	24 (23.1)	299 (19.9)	125 (19.5)	
**HER2–enriched**	301 (13.4)	10 (9.6)	202 (13.4)	89 (13.9)	
**TNBC**	395 (17.6)	23 (22.1)	258 (17.2)	114 (17.8)	
**AJCC stage at initial diagnosis (%)**					0.063
1	666 (29.6)	30 (28.8)	452 (30.1)	184 (28.8)	
2	467 (43.0)	45 (43.3)	661 (44.0)	261 (40.8)	
3	545 (24.2)	22 (21.2)	348 (23.2)	175 (27.3)	
4	63 (2.8)	7 (6.7)	36 (2.4)	19 (3.0)	
Unknown	7 (0.3)	0	6 (0.4)	1 (0.2)	
**Operation (%)**	2189 (97.4)	101 (97.7)	1470 (97.8)	618 (96.4)	0.102
**Chemotherapy (%)**	1596 (71.0)	95 (91.3)	1123 (74.7)	378 (59.0)	<0.001
**Hormone therapy (%)**	1432 (63.7)	66 (63.5)	918 (61.1)	432 (67.4)	0.087
**Brain metastasis event (%)**	164 (7.3)	19 (18.3)	109 (7.3)	36 (5.6)	<0.001
**Cumulative incidence of brain metastasis,% (95% CI)**					<0.001
1 year	1.2 (1.0–1.4)	3.0 (1.3–4.7)	1.0 (0.7–1.3)	1.2 (0.8–1.6)	
3 year	4.9 (4.4–5.4)	10.4 (7.1–13.7)	4.7 (4.1–5.3)	4.5 (3.6–5.4)	
5 year	8.2 (7.5–8.9)	20.5 (15.3–25.7)	7.8 (6.9–8.7)	7.5 (6.1–8.9)	

AJCC, American Joint Committee on Cancer; CI, confidence interval; TNBC, triple negative breast cancer.

### Cumulative Incidence of Brain Metastasis and Risk Factors for Brain Metastasis

**Figure 2:**
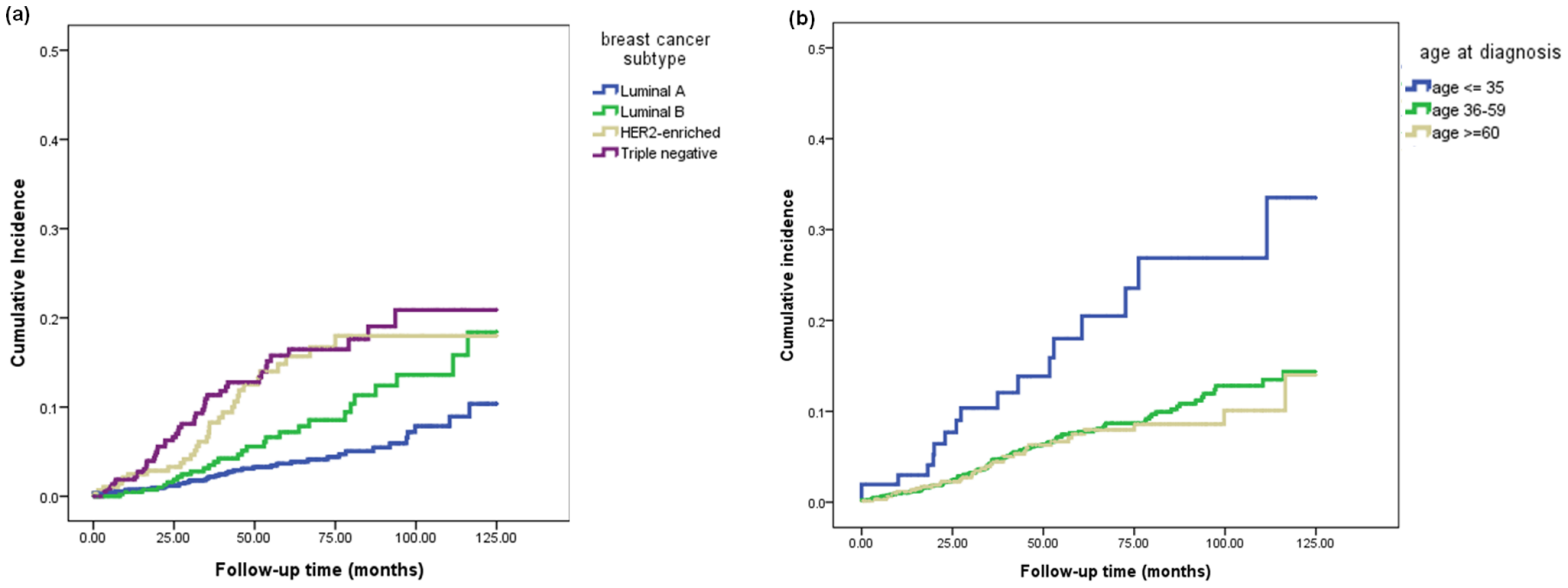
Cumulated incidence of brain metastasis according to breast cancer subtype (a), and age (b).

After a median follow-up of 54 months, 164 patients (7.3%) in this cohort developed brain metastases. In general, the estimated 5-year cumulative incidence of brain metastasis was 8.2% (95% CI: 7.5%–8.9%), and the median time from diagnosis to brain metastasis was 35.3 months (IQR 57.1–19.7 months). For patients with HER2-enriched breast cancer and TNBC, respective cumulative incidence of brain metastasis at five years was 15.7% (95% CI: 12.9%–18.5%) and 15.8% (95% CI: 13.5%–18.1%), compared with 3.7% (95% CI: 3.0%–4.4%) for luminal A type and 7.2% (95% CI: 5.6%–8.8%) for luminal B type (Table 2 and Fig. 2a). The incidence of brain metastasis in patients age 35 or younger was significantly higher than the other two age groups (Fig. 2b); the cumulative brain metastasis incidence at five years for patients age 35 or younger was 20.5% (95% CI: 15.3%–25.7%), compared with 7.8% (95% CI: 6.9%–8.7%) for patients 36–59 years and 7.5% (95% CI: 6.1%–8.9%) for patients older than 60 years.

Univariate analysis revealed that advanced stage, age below 35 at diagnosis, and non-luminal A subtypes were potential risk factors for brain metastasis (Table 4). In the multivariable Cox regression model, age 35 or younger (Hazard ratio (HR) = 2.09, 95% CI: 1.15–3.81), HER2-enriched (HR = 2.53, 95% CI: 1.57–4.07 compared with luminal A disease) and TNBC (HR = 4.42, 95% CI: 2.86–6.85 compared with luminal A disease) subtypes were independently associated with higher risk of brain metastasis (Table 4).

**Table 4:** Cox regression model for factors associated with the incidence of brain metastasis.

Factor	Univariate		Multivariate	
	Hazard ratio (95% CI)	*P*	Hazard ratio (95% CI)	*P*
**Initial AJCC stage 4 vs.1**	63.12 (33.88–117.59)	<0.001	67.07 (34.05–132.14)	<0.001
**Age at diagnosis**				
≤35 vs. ≥60	2.93 (1.67–5.14)	<0.001	2.09 (1.15–3.81)	0.016
≤35 vs. 36–59	1.13 (0.77–1.64)	0.537		–
**Biological subtype**				
Luminal B vs. Luminal A	1.76 (1.12–2.78)	0.015	1.35 (0.83–2.20)	0.231
HER2 enriched vs. Luminal A	2.82 (1.82–4.49)	<0.001	2.53 (1.57–4.07)	<0.001
TNBC vs. Luminal A	3.63 (2.41–5.48)	<0.001	4.42 (2.86–6.85)	<0.001
**Histology grade 3 vs. 1**	5.89 (1.45–23.93)	0.013	2.29 (0.54–9.80)	0.262

AJCC, American Joint Committee on Cancer; CI, confidence interval; TNBC, triple negative breast cancer.

### Analysis of Effects of Age and Biological Subtypes on Risk of Brain Metastasis

**Figure 3:**
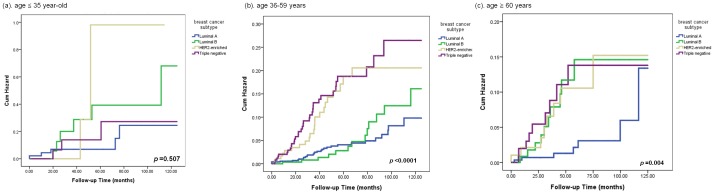
Cumulated hazard ratio of brain metastasis according to breast cancer subtype in patients aged (a) ≤35 years, (b) 36–59 years and (c) ≥60 years.

To further delineate the effects of age and breast cancer subtypes on risk and timing of brain metastasis, we analyzed cumulative incidences of brain metastasis of the different subtypes, separated by different age groups at diagnosis (Fig. 3). In patients age 35 or younger (Fig. 3a), the risk of brain metastasis for all the breast cancer subtypes was similar (*P = *0.507), suggesting that younger age independently affected risk of brain metastasis. In contrast, breast cancer subtype had a stronger impact in older patients (Fig. 3b and 3c). In patients aged 36–59 years (Fig. 3b), patients with HER2 enriched disease or TNBC, but not those with luminal B subtype, had significantly higher risks of brain metastasis compared with patients with luminal A disease (*P*<0.001). In patients older than 60 years (Fig. 3c), patients with HER2 enriched disease, or TNBC, as well as those with luminal B subtype, had significantly higher risks for brain metastasis as compared with luminal A subtype (*P = *0.004).

### Outcome for Brain Metastasis

**Figure 4:**
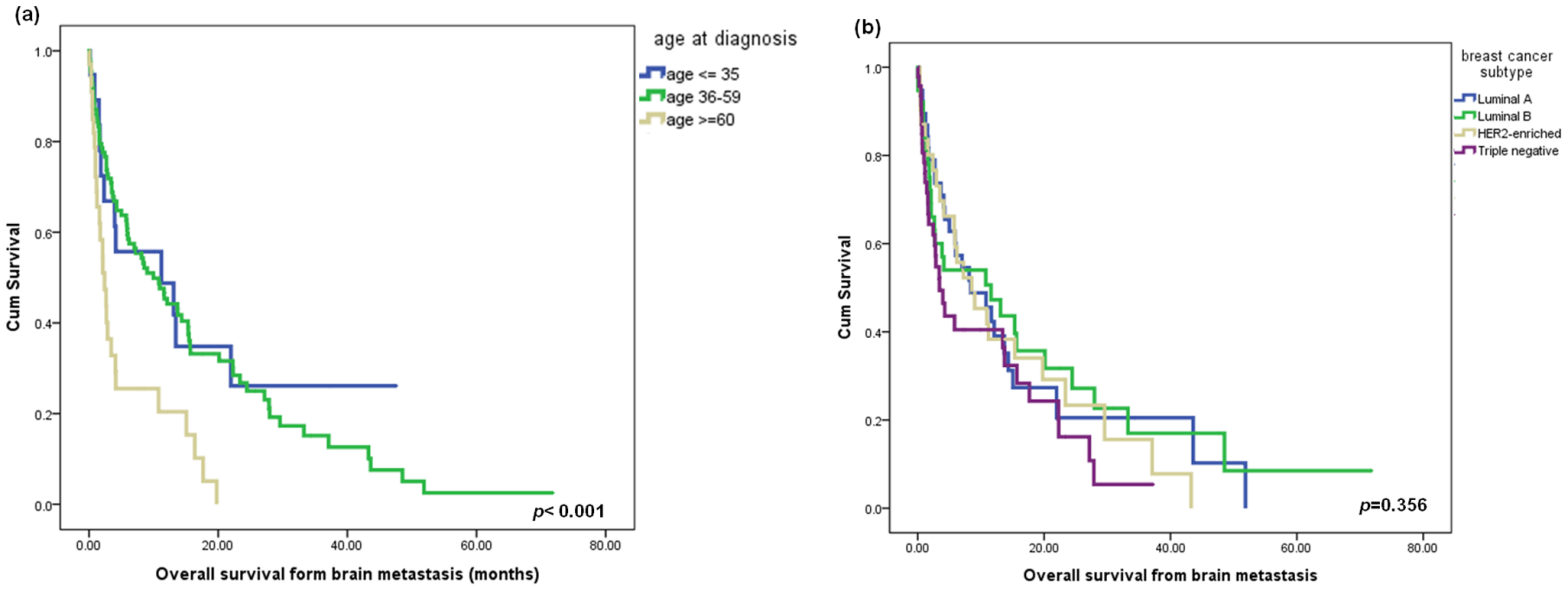
Overall survival after brain metastasis diagnosis, according to patients’ ages (a) and subtypes (b).

The majority of patients’ performance statuses were affected and more than three-quarters of patients’ Karnofsky Performance Score was ≤60 when brain metastasis was discovered (10% of patents were below 40). Subsequently, most of the patients (78.7%) received whole brain radiotherapy, thirteen patients (7.9%) received surgical resection and ten patients (6.1%) received radiosurgery. Systemic chemotherapy was given to 104 patients (63.4%). During the follow-up period, 112 (68.3%) of the 164 patients with brain metastasis died. The median OS after brain metastasis was 7.2 months (IQR 14.0–1.4 months), which was affected by age at breast cancer diagnosis (Fig. 4a), but not by breast cancer subtype (*P = *0.356; Fig. 4b).

## Discussion

Breast cancer subtype and age are well-recognized risk factors for the development of brain metastasis [4]–[6], [8], [10]. However, the effects of these two factors are usually discussed separately, and in hitherto reported data and information regarding the interplay between age and subtype has been lacking. In this study we analyzed the specific risk of brain metastasis associated with different breast cancer subtype in several age groups. In 2248 breast cancer patients with uniform treatment and follow-up, we found that young age at diagnosis (age ≤35) was indicative of increased risk of brain metastasis independent of breast cancer subtype. In contrast, risk of brain metastasis was strongly associated with breast cancer subtype in older patients, and patients with HER2-enriched and TNBC phenotypes had a higher risk of brain metastasis than patients with hormone receptor-positive disease.

According to the “seed and soil” theory proposed by Paget et al. [16], the metastatic behavior of tumors may be correlated to certain features of the primary tumor that result in “tissue tropism”. Several characteristics of patients and primary breast tumors were previously found to be associated with higher risk of brain metastasis. Young age, ER negativity, and HER2 overexpression are consistently reported to be associated with higher risk of brain metastasis in most studies [4]–[6], [7], [10], [17]–[18]. Likewise, our study found that earlier onset of breast cancer (i.e., breast cancer diagnosis before the age of 35), HER-2-enriched and TNBC disease were significantly associated with higher incidence of brain metastasis (Table 4).

In our study, we further observed the impact of different subtypes on each age group. Contrasting with older patients, HER2-enriched disease and TNBC shared a similar risk of brain metastasis to hormone positive disease among patients whose disease was diagnosed before the age of 35. Our results suggested that the four biological subtypes of breast cancer might be similarly aggressive with respect to metastasis to the brain in young patients, and the data suggested that the better prognosis usually associated with hormone receptor-positive disease is diminished or reversed in younger breast cancer patients. In a cohort of 1320 breast cancer patients reported by Saghir et al., worse outcome was seen in younger patients, especially those with hormone receptor-positive disease [19]. Another retrospective study also showed that disease-free survival after adjuvant chemotherapy in younger patients with ER-positive tumors was significantly worse than patients with ER-negative disease; whereas outcomes for older patients were not affected by ER status. [20] Although different end-points were used (disease progression and the development of brain metastasis), results of these two studies and our study suggest that breast cancer has unique characteristics in young patients. Anders *et al.* described different gene expression profiles in tumors from younger women. [21] They found lower expression of ER alpha and beta mRNA, with higher expression of HER-2 and epidermal growth factor receptor (EGFR) compared to older patients. [21] Moreover, elevated EGFR expression was associated with brain metastasis [22], [23]. This finding could help to explain the disease course that we observed in young patients.

In our study, 112 (68.3%) among the 164 patients with brain metastasis died of breast cancer according to careful chart review of their death certificates. Censored survival data of patients post brain metastasis diagnosis would be more valuable if cause of death could be ascribed specifically to brain metastasis. Our data showed that outcomes after brain metastasis were not significantly associated with any breast cancer subtype. Niwińska et al. reported a similar finding [24], while other studies implied worse prognoses for patients with ER–, HER2+ and TNBC [25], [26]. The discrepancy between our study and others might be explained by relatively worse performance status at brain metastasis and fewer patients who were eligible for intensive surgical intervention for their brain metastasis. In line with previous study results [26], [27], age at diagnosis was significantly associated with outcome after brain metastasis; worse outcome was seen in patients older than 60 years, whose performance status was usually poor and had limited clinical tolerability for intensive treatment. [27], [28].

Our study has several potential limitations. First, the number of young patients included in this study was relatively small, reflecting the intrinsic rarity of this subgroup. Second, the classification of breast cancer subtypes was determined by expression of ER, PR, HER2, and pathological grade. Accordingly, our findings might not apply to subtypes determined by genotype profiling methods. However, studies increasingly suggest that these two methods strongly correlate [11], [12]. The retrospective and single-center design of this trial might lead to potential selection bias. Moreover, the incidence of occult brain metastases, which might have skewed the significant differences observed for the risks of brain metastasis development, could not be assessed by the current study as no patients were autopsied, and CNS imaging was not routinely performed. Nevertheless, the follow up for each patient was consistent in the current study regardless of each patient’s age. Previous reports showed that occult brain metastases are more likely to occur in patients with metastatic breast cancer [30]–[31], and the distribution of metastatic breast cancer in the age <35 versus >60 groups did not differ significantly (Table 3). Moreover, Miller et al. screened 155 patients with metastatic breast cancer and identified 15% of patients with asymptomatic brain metastases visible on MRI or CT images [30]. In their study, age was not a predictive factor for occult brain metastases. Similarly, Kaplan et al. prospectively enrolled 96 HER2+ breast cancer patients (with or without metastases at enrolment) and screened for brain metastasis by MRI [31]. They found 11 (11.5%) occult brain metastases and age had no impact in the development of occult brain metastases [31]. It remains unclear whether age is also a significant risk factor or predictive indicator of occult brain metastases, and further studies are needed.

In conclusion, this study provides data about brain metastasis risk in breast cancer patients according to breast cancer subtype and age group, and is the first to show the varied effect of subtypes among different age groups. Data from this study suggest that in younger patients, risk of brain metastasis is independent of biological subtype, while elderly patients with ER+ disease have significantly lower risk of brain metastasis compared with those with HER-2 enriched and TNBC. Our findings enhance understanding of how age and breast cancer subtype affect brain metastasis. These characteristics should be considered when developing surveillance and prevention strategies for patients.
