# Correction: Effect of Age and Biological Subtype on the Risk and Timing of Brain Metastasis in Breast Cancer Patients

**DOI:** 10.1371/journal.pone.0100460

**Published:** 2014-06-13

**Authors:** The PLOS ONE Staff

The following information is missing from the Funding section: This study was also supported by a grant from the Ministry of Health and Welfare, Grant number MOHW103-TD-B-111-02.

## References

[1] Hung M-H, Liu C-Y, Shiau C-Y, Hsu C-Y, Tsai Y-F, et al (2014) Effect of Age and Biological Subtype on the Risk and Timing of Brain Metastasis in Breast Cancer Patients. PLoS ONE 9(2): e89389 doi:10.1371/journal.pone.0089389 24586742 10.1371/journal.pone.0089389 PMC3933537

